# Sexual dimorphism in size and shape of the head in the sea snake *Emydocephalus annulatus* (Hydrophiinae, Elapidae)

**DOI:** 10.1038/s41598-021-99113-2

**Published:** 2021-10-08

**Authors:** Richard Shine, Claire Goiran

**Affiliations:** 1 Department of Biological Sciences, Macquarie University, Sydney, NSW 2109 Australia; 2 Labex Corail & ISEA Université de la Nouvelle-Calédonie, BP R4, 98851 Nouméa cedex, New Caledonia

**Keywords:** Zoology, Behavioural ecology, Evolutionary ecology, Tropical ecology, Evolution, Sexual selection

## Abstract

In snakes, divergence in head size between the sexes has been interpreted as an adaptation to intersexual niche divergence. By overcoming gape-limitation, a larger head enables snakes of one sex to ingest larger prey items. Under this hypothesis, we do not expect a species that consumes only tiny prey items to exhibit sex differences in relative head size, or to show empirical links between relative head size and fitness-relevant traits such as growth and fecundity. Our field studies on the sea snake *Emydocephalus annulatus* falsify these predictions. Although these snakes feed exclusively on fish eggs, the heads of female snakes are longer and wider than those of males at the same body length. Individuals with wider heads grew more rapidly, reproduced more often, and produced larger litters. Thus, head shape can affect fitness and can diverge between the sexes even without gape-limitation. Head size and shape may facilitate other aspects of feeding (such as the ability to scrape eggs off coral) and locomotion (hydrodynamics); and a smaller head may advantage the sex that is more mobile, and that obtains its prey in narrow crevices rather than in more exposed situations (i.e., males).

## Introduction

Adult males and females of the same species often differ from each other in a wide range of phenotypic traits, not simply in characteristics directly related to the production and release of gametes^1,2^. For example, males and females may attain different body sizes, differ in body proportions and display structures (such as dewlaps and manes), and interact differently with conspecifics, competitors and predators^3,4^. An extensive literature interprets such divergences as the result of (a) sexual selection, whereby differing reproductive roles favour phenotypes in each sex, and (b) fecundity selection, whereby larger body size increases reproductive output in females^5,6^. The resultant sexual dimorphism may result in ecological differences, further amplifying or reducing dimorphism^7,8^. Thus, for example, males might evolve to be the larger sex because of advantages in male–male rivalry; but that larger size enables them to overpower and consume larger types of prey, favouring adaptations to deal more effectively with the novel prey type^9^. In the extreme case, the larger sex might obtain access to an abundant prey resource inaccessible to the smaller sex, resulting in the evolution of a greater disparity in mean adult body sizes^10^.

Although the “ecological causation” hypothesis is plausible, evidence is limited because it is difficult to distinguish between sexually-selected dimorphism and ecologically-driven dimorphism. Thus, for example, males have relatively larger heads than females in many species of lizards, and consume larger prey items^11^. But males also use their jaws to fight with each other, and larger head size may enhance success in combat^9,12^. In that scenario, the sex divergence in diets may be a consequence of sexually-selected divergence in head morphology, rather than a selective force for the evolution of that divergence. To confidently identify cases of ecological causation for sexual dimorphism, we need a species in which the trophic structures do not play a role in sexual conflict or social signalling^8^. Snakes provide an excellent model in this respect. Even in the minority of snake species in which rival males engage in physical battles for mating opportunities, the bouts are highly ritualised and (except in a few species) do not include biting^13,14^. In contrast, head dimensions can constrain prey choice: many snakes consume very large prey items, and they cannot tear such items apart to reduce ingestible size^15^. Because a snake’s head size is likely to affect its ability to consume large prey, but not to conquer rival conspecifics, the widespread occurrence of sex differences in relative head size in snakes supports the hypothesis that sexual dimorphism can be modified by niche divergence between the sexes^13^.

In the present paper, we provide data on a snake species (*Emydocephalus annulatus*) that does not conform to the assumptions of the scenario outlined above. Unusually, individuals of this species feed only on the eggs produced by small fishes (gobies, blennies, damselfish) on coral reefs^16^. As a result, even a neonatal snake (around 300 mm snout-vent length [SVL], 13 mm head length) can ingest any available egg (< 1.5 mm diameter; eggs are consumed singly, not as a clump)^17^. Because gape-limitation does not apply, we might expect male and female snakes of this species to have similarly-sized heads relative to body size. Also, we might expect that variation among individuals in relative head size will not affect fitness-relevant processes such as rates of growth and reproduction. Data from our long-running (to date, 18-year) field study falsify both of those predictions.

## Results

### Sexual dimorphism

**Figure 1:**
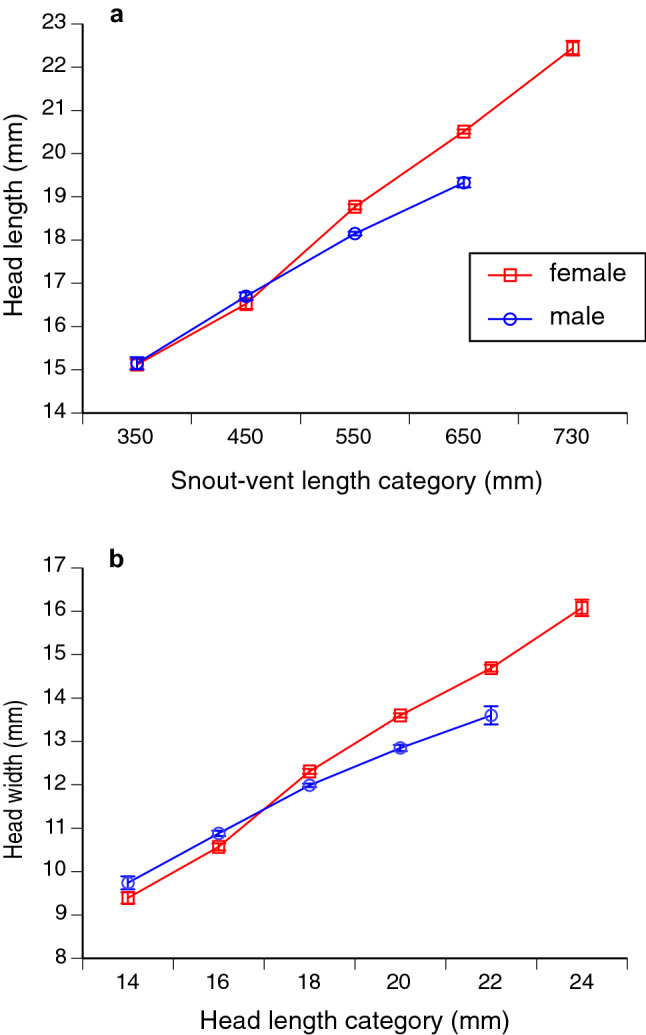
Sexual dimorphism in the sea snake *Emydocephalus annulatus*, in terms of (**a**) head length relative to snout-vent length (SVL); and (**b**) head width relative to head length. Variables on the x-axis are combined into categories to facilitate visualisation of trends; each interval of SVL or head length shows mean value and associated standard error.

The length of a snake’s head was affected by an interaction between sex and SVL (F_1,2204_ = 19.82, P < 0.0001); females exhibited increasingly larger heads than males at larger body sizes (see Fig. 1a). The same pattern was seen for head widths (sex × SVL, F_1,2498_ = 34.45, P < 0.0001; Fig. 1b).

### Effect of relative head size on rate of growth

**Figure 2:**
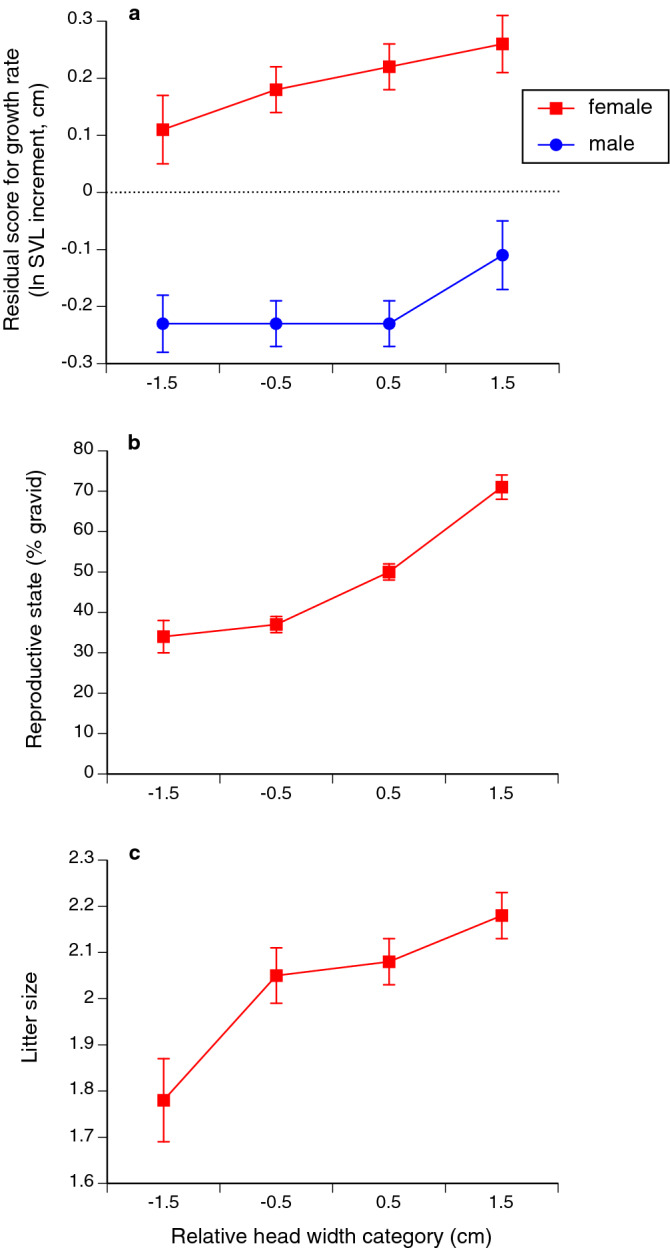
Relationships between the head shape (width relative to length) of a turtle-headed sea snake *Emydocephalus annulatus*, and (**a**) the snake’s growth rate (increment in snout-vent length [SVL] per year), (**b**) reproductive state (gravid or not) and (**c**) litter size. Relative head-width values are combined into categories to facilitate visualisation of trends; each interval of relative head length shows mean value and associated standard error for the relevant variable.

In both sexes, growth rates were higher in snakes that were smaller in body size (SVL effect for males, F_1,578.7_ = 395.55, P < 0.0001; SVL effect for females, F_1,554.8_ = 230.00, P < 0.0001) and in snakes with relatively wider heads (males, F_1,575.1_ = 14.40, P < 0.0002; females, F_1,597_ = 10.00, P < 0.002; see Fig. 2a). Relative head length was not significantly linked to growth rate in either sex (males, F_1,592.2_ = 2.32, P = 0.13; females, F_1,629.9_ = 0.001, P = 0.96).

### Effect of relative head size on reproductive frequency

A snake was more likely to be gravid when captured if it was large (SVL effect, F_1,622_ = 80.08, P < 0.0001) and had a wide head (F_1,622_ = 5.37, P < 0.021; see Fig. 2b). Relative head length was not significantly linked to reproductive state (F_1,622_ = 1.85, P = 0.17).

### Effect of relative head size on litter size

The number of offspring in a litter was higher for females that were larger (SVL effect, F_1,494.1_ = 4.74, P < 0.03) and had wider heads (F_1,564.7_ = 15.34, P < 0.0001; see Fig. 2c), but the effect of increased head length on increased litter size fell short of statistical significance (F_1,566.7_ = 3.74, P = 0.054).

## Discussion

The heads of female turtle-headed sea snakes are larger than those of males, both in terms of size (length relative to snake body length), and shape (head width relative to head length; Fig. 1). Additionally, snakes with relatively wider heads exhibited more rapid growth, reproduced more frequently, and had larger litters when they did so (Fig. 2). Those patterns indicate that gape-limitation (i.e., head size constrains maximal ingestible prey size) is not necessary for sex-based differences in trophic structures to evolve, or for head dimensions to affect fitness.

Sexual dimorphism in relative head size is labile in snakes, both within and among species^13^. Within the aipysurine lineage of sea snakes, heads of females are larger than those of males in *Aipysurus laevis*^18^ and *E. annulatus* (present study), but males have larger heads than females in *A. eydouxii*^19^. The study on *A. eydouxii* was based on skull lengths (from the rostral point of the premaxilla to the most caudal projection of the cranium) of preserved specimens, rather than measurements of live animals as in the case of the other two species. However, the correlation between these two measures likely is high. Future work could usefully explore that variation in more detail, by obtaining data on other aipysurine species, by sampling multiple populations within species, and by evaluating whether gape-limitation applies in each taxon.

Two mechanisms (or a combination thereof) might explain the sex-based divergence in head dimensions in *E. annulatus*. Experimental studies on captive snakes of several species have shown that head size can be affected by phenotypic plasticity as well as by canalised adaptation^20,21^. Such flexibility occurs in *Notechis scutatus*, an Australian terrestrial elapid closely related to the ancestral hydrophiines that made the transition to aquatic life^22,23^. Interestingly, individuals from some populations of *N. scutatus* develop large heads only if fed on larger prey items, whereas individuals from other populations exhibit large heads regardless of feeding regime^23^. Although the prey items consumed by *E. annulatus* are too small to favour head enlargement through the repeated physical stress of ingesting large prey, other components of feeding behaviour (e.g., scraping against hard substrates to detach eggs, sucking those eggs into the mouth^24^) might impose similar pressures, and hence modify trophic morphology. Alternatively or additionally, natural selection may have favoured larger heads in female snakes (i.e., a canalised rather than phenotypically plastic response) for the same kinds of biomechanical reasons.

Our mark-recapture studies on *E. annulatus* show that females grow faster than males, and they continue growing for longer^25^. A larger head apparently facilitates faster growth (Fig. 2a). Also, despite biennial reproduction, the energy allocation to reproduction for a female turtle-headed sea snake is high^25^. A larger head was linked to higher reproductive output (Fig. 2b,c), perhaps reflecting a functional link between larger head size and higher rate of feeding. If the energy requirements for growth and reproduction in females exceed the metabolic cost of mate-searching by males, then females may be under more intense selection to optimise head morphology with respect to foraging. In contrast, the evolution of head dimensions of males may be influenced by effectiveness in other functions. For example, a smaller head is more hydrodynamically efficient^26^, perhaps conferring advantages during the frequent, extensive and rapid mate-searching behaviour exhibited by males^27^ or when accessing prey within narrow crevices^28^.

**Figure 3:**
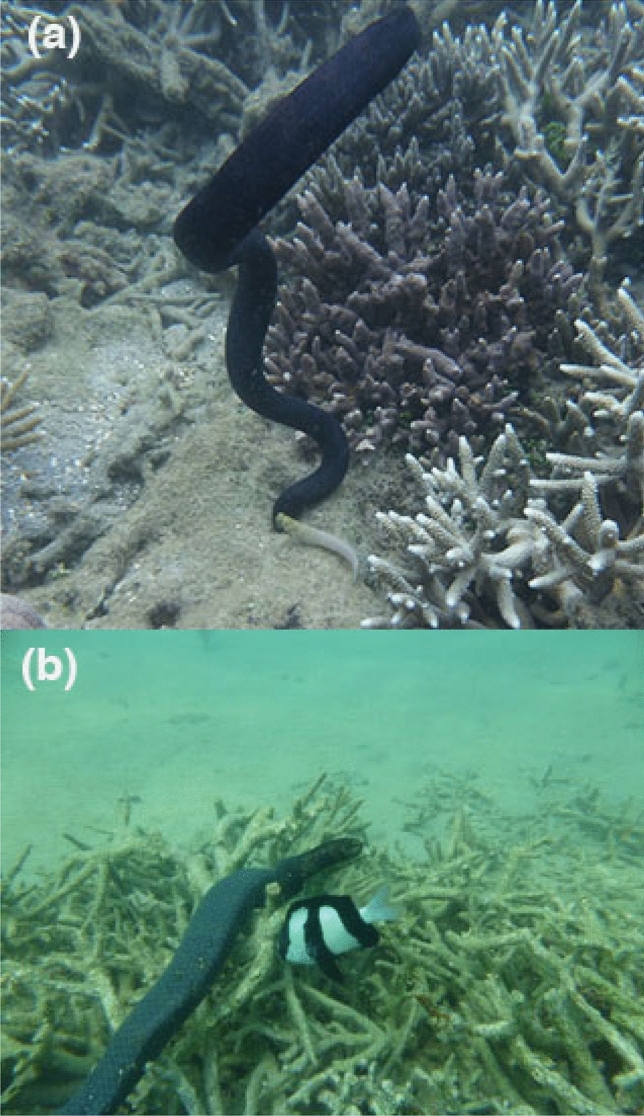
The only prey taken by Turtle-headed sea snakes, *Emydocephalus annulatus*, are the eggs of small demersal-spawning fishes. The snakes obtain some prey from burrows, and some from more open situations. Image (**a**) shows a snake with its head down the burrow of a blenny (*Salarius fasciatus*), consuming eggs, whereas image (**b**) shows a snake scraping eggs from a damselfish (*Dascyllus aruanus*) nest on exposed branching coral. In both cases, adult fish (parents of the eggs being consumed) ineffectually attack the snakes. Photographs by C. Goiran.

Crevice-foraging may be more important for male snakes than for females. Female *E. annulatus* more frequently consume the eggs of winter-spawning damselfish than do males (which focus on mate-searching at this time of year); both sexes consume the eggs of gobies and blennies in summer^16^. The latter prey items are hidden within burrows and crevices in the substrate, requiring snakes to force their heads inside narrow openings to reach the food (see Fig. 3). In contrast, damselfish nests often are laid on more exposed sites such as branching coral and open boulders^29^, such that a snake’s head size does not affect its access to the prey. That sex difference in prey types may relax selection to minimise head size in female snakes, prioritising the higher rates of ingestion putatively achievable by snakes with larger heads.

Importantly, our data do not falsify the hypothesis that gape-limitation has played an important role in generating head-size dimorphism in snakes^13^, but we show that this kind of dimorphism can evolve in the absence of that selective pressure. The size and shape of an individual’s head may have multiple functionally significant effects, including sensory ability (e.g., eye size), information processing (e.g., brain size), social signalling (visual stimulus in displays), bite strength (influencing fighting and antipredator tactics) and the ability to move the head rapidly and efficiently through water (hydrodynamics of striking and locomotion). The trade-off between maximum ingestible prey size and ability to access narrow crevices is an important functional consequence of variation in relative head size, but it is not the only significant such consequence. Studying a snake species in which jaw dimensions do not constrain prey size illustrates the value of “unusual” species in testing general hypotheses, and highlights the importance of exploring multiple functional consequences of variation in sexually dimorphic traits.

## Methods

### Study species and area

The turtle-headed sea snake (*Emydocephalus annulatus*) is a stout-bodied medium-sized snake belonging to the aipysurine lineage of hydrophiine elapids^28^ (see Fig. 3). Widely distributed in shallow coral-reef habitats of the Indopacific region, extensive studies (including, genetic analyses of ingested items) have shown that the species feeds only on fish eggs, which are scraped off the substrate with the enlarged labial scales that line the upper jaw^16,24^. Reflecting this unusual diet, the snakes have vestigial venom glands and fangs^30^. Females attain larger sizes than males (mean adult snout-vent length [SVL] = 600 vs. 550 mm; mean adult mass = 200 vs. 130 g)^25^. Adult females reproduce on an approximately biennial schedule, producing a litter of one to three offspring that attain sexual maturity in 2–3 years^25^. Although all individuals eat fish eggs, females cease feeding during gestation in late summer and males cease feeding during the mating season in winter; as a result, the sexes differ in the fish species whose eggs they consume (more damselfish eggs in winter, more gobies and blennies in summer)^16^. Recapture records and genetic data show that snakes are highly philopatric^31^.

We captured, marked and recaptured snakes at two small bays (Baie des Citrons and Anse Vata) beside the city of Noumea in the Pacific nation of New Caledonia. Water depth ranges from 0 to 3 m, with a complex substrate mosaic dominated by live coral, coral rubble, boulders and sand^16,32^. A snake’s sex and body size are not linked to its habitat use^33^.

### Methods

Every January from 2004 to 2021, we snorkelled through the study sites at least 20 times in total (30–60 min per session). All snakes seen were captured by hand, and processed at a nearby laboratory (measured, weighed, assessed for sex, palpated for pregnancy and litter size; implanted with a microchip, and released at the site of capture) (see Ref.^25^ for detailed methods). We determined sex based on tail shape and scale rugosity^27^. We used calipers to measure the length of the head (tip of snout to rear end of quadrate-articular projection of the lower jaw) and maximum width of the head. We did not include the rostral spine of males in our measurement of head length.

We only took data from a snake once per year, at its first capture in that period, but many snakes were recaptured in subsequent years. In total, we have data on 1293 individual snakes (662 males, 631 females) captured a total of 3012 times over the period January 2004 to January 2021 (Supplementary Information).

### Ethics statement

The research was conducted under animal ethics approval 2015/880 (University of Sydney) and permit 3252-17/ARR/DENV (Province Sud, New Caledonia). All procedures involving animals were carried out in accordance with relevant guidelines and regulations (including ARRIVE guidelines).

### Statistical analyses

We tested for normality and variance homogeneity before conducting tests, and ln-transformed data when that procedure improved normality or (for calculation of residual scores) linearity of regressions. We used the programs JMP Pro 15 and SAS 9.4, and included individual snake ID # as a random factor in all analyses to account for repeated measures on some animals. To examine sex differences in head dimensions, we conducted two ANCOVAs. The first had head length as the dependent variable and SVL as the covariate, with independent variables being sex, and the interaction between sex and SVL (plus snake ID as a random factor). The second ANCOVA had a similar structure but with head width as the dependent variable, and head length rather than SVL as the covariate.

To evaluate influences on growth rate, the ln-transformed value of the SVL increment between successive years was used as the dependent variable in an ANOVA with the independent variables being SVL, head width and head length (and individual snake ID, a random factor). Because males and females grow at different rates^25^, we conducted those analyses separately for each sex. The same ANOVA design was used to examine variation in fecundity among females, but with litter size as the dependent variable. To explore effects of head dimensions on reproductive state (pregnant vs. non-pregnant), we used nominal logistic regression with reproductive state as the dependent variable; as before, the independent variables were SVL, head length and head width (and individual snake ID).

To illustrate trends for graphical presentation, we obtained an index of relative head width by regressing head width against head length, and took residual scores (henceforth, “relative head width”). To obtain a size-independent index of growth rates, we regressed annual increments in body size (SVL) between successive captures against initial SVL; the residual scores from that regression offer an index of growth rate compared to the expected increment for an animal of that size.

## Supplementary Information

Supplementary Video 1.

Supplementary Legends.

## Data Availability

Data are available in the Dryad Digital Repository at https://doi.org/10.5061/dryad.b2rbnzsg3.
