# Supplementary material for: Sexual dimorphism in size and shape of the head in the sea snake Emydocephalus annulatus (Hydrophiinae, Elapidae)
Source: Sci Rep. 2021 Oct 8;11:20026. doi: 10.1038/s41598-021-99113-2 (PMC8501056; doi:10.1038/s41598-021-99113-2)
Supplement: Supplementary file 2 — Supplementary Legends. [file 41598_2021_99113_MOESM2_ESM.docx]

**Supplementary information**

**Supplementary Video S1.** A female turtle-headed sea snake *Emydocephalus annulatus* scraping eggs from a damselfish (*Dascyllus aruanus*) nest on exposed branching coral while adult fish (parents of the eggs being consumed) attack the snake. Video from the Baie des Citrons by C. Goiran.
